# A Novel Index System for Assessing Ventricular-Vascular Coupling

**DOI:** 10.31083/j.rcm2410282

**Published:** 2023-10-08

**Authors:** Lingheng Wu, Mengjiao Zhang, Jianxiong Chen, Lin Jin, Cuiqin Shen, Jiali Sun, Xianghong Luo, Zhaojun Li, Lianfang Du

**Affiliations:** ^1^Department of Ultrasound, Shanghai General Hospital of Nanjing Medical University, 200080 Shanghai, China; ^2^Department of Medical Imaging, Weifang Medical University, 261053 Weifang, Shandong, China; ^3^Department of Ultrasound, Guanghua Hospital Affiliated to Shanghai University of Traditional Chinese Medicine, 200052 Shanghai, China; ^4^Department of Ultrasound, Jiading Branch of Shanghai General Hospital, Shanghai Jiaotong University School of Medicine, 201812 Shanghai, China; ^5^Department of Echocardiography, Shanghai General Hospital, Shanghai Jiaotong University School of Medicine, 200080 Shanghai, China; ^6^Department of Ultrasound, Shanghai General Hospital, Shanghai Jiaotong University School of Medicine, 200080 Shanghai, China

**Keywords:** echocardiography, ventricular-vascular coupling, arterial velocity pulse index, left ventricular global longitudinal strain, age

## Abstract

**Background:**

To explore the value of a novel ventricular-vascular coupling index (VVI) system in relation to age, gender and body mass index (BMI).

**Methods:**

A total of 239 volunteers with single-center and cross-sectional health screening were enrolled in the study. Subjects were divided according to age (young [18–44 years], middle-age [45–59 years], old [60–80 years]), gender (male, female), and BMI (overweight/obese [BMI ≥24], control [BMI <24]). The left ventricle end-diastolic volume (LVEDV) and left ventricle end-systolic volume (LVESV) provided the left ventricular structure index, while the TDI *$e^{\prime }$* provided the functional index. Also derived from routine echocardiography were the effective arterial elastance (Ea), left ventricular end-systolic elastance (Ees), and VVI. The novel VVI systems were arterial velocity pulse index (AVI), left ventricular global longitudinal strain (LVGLS), and the AVI to LVGLS ratio (AVI/LVGLS).

**Results:**

(1) Middle-age and elderly subjects had higher Ea and lower LVGLS compared to young subjects. AVI and AVI/LVGLS increased progressively from young to middle-age to old subjects. (2) Females had higher Ea, Ees and LVGLS than male subjects. No significant differences in AVI and AVI/LVGLS were observed between males and females. (3) No significant differences in Ea, Ees, VVI, AVI, LVGLS and AVI/LVGLS were observed between the overweight/obese and control groups. (4) AVI/LVGLS was negatively correlated with LVEDV and LVESV and with TDI *$e^{\prime }$*. LVEDV, LVESV and TDI *$e^{\prime }$* were independent predictors of AVI/LVGLS. (5) The diagnostic performance of AVI/LVGLS was higher than that of VVI in the young and middle-age groups. The diagnostic efficacy of AVI/LVGLS was higher than that of VVI in the young and old groups, and the diagnostic efficacy of AVI was higher than that of Ea. The difference in diagnostic efficacy between LVGLS and Ees was not statistically significant. The differences in diagnostic efficacy between AVI/LVGLS and VVI, AVI and Ea, and LVGLS and Ees were not statistically significant in the middle-age and old groups.

**Conclusions:**

The novel index system of ventricular-vascular coupling described here (AVI, LVGLS, and AVI/LVGLS) was more effective than traditional indexes in detecting differences in cardiovascular function between different ages groups.

**Clinical Trial Registration:**

The study protocol was registered on the official website of China Clinical Trial Registration Center (ChiCTR2000035937).

## 1. Introduction

One of the most significant determinants of cardiovascular function is ventricular-vascular coupling. This term refers to the connection between the left ventricle and the arterial system, which is essential for measuring the energy efficiency of the left ventricle. The ventricular-vascular coupling of the heart maximized the energy efficiency [1]. Moreover, ventricular-vascular coupling is a potential therapeutic target for enhancing the performance of both the heart and vascular system and thus delaying the onset of heart failure [2]. The assessment of ventricular-vascular coupling includes several index components, such as effective arterial elastance (Ea), which indicates systemic arterial function, left ventricular end-systolic elastance (Ees), which reflects myocardial systolic function, and the ratio of Ea to Ees (Ea/Ees), which is known as the ventricular-vascular coupling index (VVI) [3].

Sunagawa *et al*. [4] first measured the “left ventricular end-systolic pressure-volume” curve by using cardiac catheters in animal studies to calculate Ea, Ees and VVI, thereby allowing evaluation of ventricular-vascular coupling. Their findings, later corroborated by other researchers, showed that ventricular-vascular decoupling was earlier than the onset of arterial or cardiac pathogenesis [5]. However, the invasive nature of this method limited its clinical application. It was suggested that the VVI obtained by deducing formula based on echocardiography results had good concordance with the cardiac catheters [6]. The mechanically explainable optimal cardiac work efficiency is often used [7]. However, the VVI, defined as [(left ventricle end-systolic pressure (LVESP)/stroke volume (SV))/(LVESP/left ventricle end-systolic volum (LVESV)), LVESP = 0.9 × systolic blood pressure (SBP)], equivalent to 1/(left ventricular ejection fraction [LVEF] – 1) and obtained by echocardiography only reflects the systolic function of the ventricle and not the arterial function directly, since the measured values of Ea and Ees are homologous. In addition, the factors of age, gender and body mass index (BMI) are all implicated in arterial and cardiac degeneration [8], with age being the main factor [9]. Arterial and ventricular stiffness increase with age, whereas the VVI remains relatively steady. VVI attenuates synchronous changes to ventricular and arterial functions [10]. Therefore, it is important to identify more sensitive markers that can independently assess arterial and ventricular function, overcome the homology issue between Ea and Ees, and systematically evaluate VVI. Trambaiolo *et al*. [11] described a simple and non-invasive method to assess ventricular-vascular coupling based on echocardiography. This method uses a special computer to determine Ees, thus allowing VVI to be obtained without relying on LVEF alone and making it suitable for use in the intensive care unit (ICU) setting. The ratio of pulse wave velocity (PWV), an indication of arterial stiffness, to global longitudinal strains (LVGLS) of the left ventricle, which reflects arterial stiffness and myocardial deformation through mechanical coupling, is recognized as a marker of ventricular-vascular coupling with promising clinical applications [12]. PWV is widely used as the “gold standard” measure of arterial stiffness, while LVGLS acquired with two-dimensional speckle tracking imaging can be used to evaluate early and subclinical cardiac dysfunction [5]. As PWV increases, arterial stiffness also increases and this can affect myocardial deformation and LVGLS through mechanical coupling [12]. Compared with Ea and Ees obtained by traditional echocardiography, PWV and LVGLS are two independent indicators used for evaluating arterial and cardiac function. Therefore, PWV/LVGLS may be more sensitive at evaluating ventricular-vascular coupling than traditional VVI [5]. However, PWV is challenging to apply for the large-scale screening of community populations due to its long measurement time, high operator dependence, and many environmental factors [13].

In recent years, the arterial velocity pulse index (AVI) has emerged as a novel index of arterial stiffness. AVI has attracted considerable research attention as a possible alternative to PWV [14]. It is calculated non-invasively using cuff oscillometry, which quantitatively analyzes the oscillatory waves of proximal brachial artery cuff pressure to assess arterial elastance. AVI has been found to reflect central arterial pressure, and impaired AVI may indicate an increased cardiac load [15]. Earlier prospective studies showed that AVI is a powerful predictor of cardiovascular events in patients with hypertension combined with heart failure with preserved ejection fraction (HFpEF), and may improve their risk prediction [16]. Our previous research based on a large sample size found that AVI presents a “J” pattern with age, whereby it decreases before the age of 20 years and increases thereafter. This suggests a process of development and maturity of arterial elastance before the age of 20 [17]. Furthermore, AVI has been found to reflect gender- and age-related differences in arterial elastance, and to independently predict the risk of cardiovascular events over the following 10 years [18]. AVI also has the advantages of simple operation, low operator dependence, and good reproducibility of measurement, thus making it a promising tool for large-scale population monitoring or screening [19].

In this study, we hypothesized that a novel cardiovascular coupling index system consisting of AVI, LVGLS and AVI/LVGLS provides a more sensitive reflection of the impact of age on cardiovascular function compared to traditional, echocardiography-derived Ea, Ees and VVI. Age, gender and BMI are known to influence both arterial and cardiac function. Hence, the aim of this study was therefore to investigate the effect of age, gender and BMI on ventricular-vascular coupling using the new index system in a healthy volunteer population. The findings of this study should provide novel perspectives for the systematic evaluation of cardiac performance.

## 2. Materials and Methods 

### 2.1 Subjects 

A single-center, cross-sectional study design was used. Healthy volunteers from the medical examination center of Jiading Branch of Shanghai General Hospital between January 2022 and January 2023 were selected as the study subjects. Demographic information including age, gender, height and weight was recorded for each participant using electronic data capture technology. BMI for each participant was calculated as weight (kg)/square height ($m^{2}$), and BSA was defined as 0.0061 × height (cm) + 0.0128 × weight (kg) – 0.1529. Healthy volunteers aged 18–80 years with complete echocardiographic data were recruited to the study. Subjects with the following conditions were excluded: (1) age <18 years or >80 years; (2) endocrine system diseases such as diabetes mellitus or hyperthyroidism; (3) hypertension with systolic arterial pressure >140 mmHg and/or diastolic arterial pressure >90 mmHg and a value of 125/80 mmHg on 24-hour ambulatory blood pressure monitoring; (4) hematological disease, malignant tumor, or serious liver, kidney and lung disease; (5) a previous history of cardiovascular disease; (6) patients with atrial fibrillation; (7) congenital heart disease. All subjects were divided into different groups according to age, gender and BMI to explore the effect of these variables on the novel index of ventricular-vascular coupling. Specifically, patients were stratified into three age groups according to the World Health Organization age criteria. These were defined as the young group (<44 years), the middle-age group (45 years ≤ age < 60 years) and the old group (≥60 years) [20]. Gender groups were defined as male or female, and BMI groups as overweight/obese (BMI ≥24) or controls (BMI <24) according to The Chinese National Health Commission weight criteria [21]. The study protocol was approved by the Ethics Committee of Shanghai General Hospital (2021KY057) and registered on the official website of China Clinical Trial Registration Center (ChiCTR2000035937). All participants provided written informed consent.

### 2.2 Ventricular-Vascular Coupling Based on Echocardiography

A commercially available system (EPIQ7, Philips, S5 probe, Amsterdam, Netherlands) with a frequency of 1–5 MHz, a frame frequency of ≥60 frames/sec, and an inspection depth of 13–16 cm was used to acquire the original images. The electrocardiogram was connected and recorded synchronously. Prior to the measurements, subjects were rested for more than 5 minutes to ensure stable hemodynamic conditions. The left ventricular end-diastolic diameter (LVEDD) and left ventricular end-systolic diameter (LVESD) were obtained using M-mode technique. Pulsed Doppler was used in an apical 4-chamber view to measure early mitral orifice diastolic flow velocity (E) and late mitral orifice diastolic flow velocity (A). Tissue Doppler imaging was employed to obtain the motion velocity of mitral annulus (TDI *$e^{\prime }$*) of left ventricular lateral wall in early diastole. The index of left ventricular diastolic function E/A (normal cutoff values: late *$e^{\prime }$* <10 cm/sec, E/A <0.8) was calculated [22]. Left ventricular end-diastolic volume (LVEDV), left ventricular end-systolic volume (LVESV), stroke volume (SV) and LVEF were measured using the biplane Simpson method.

Mean arterial pressure (MAP) was calculated as [(2 × diastolic blood pressure (DBP)) + SBP/3] [23]. The left ventricular mass (LVM) was calculated using the formula 0.8 × 1.04 × [${\mathrm{(LVEDD + IVS + LVPW)}}^{3}$ – ${\mathrm{LVEDD}}^{3}$] + 0.6. The left ventricular end-systolic pressure (LVESP) was calculated as 0.9 × SBP, the Ees was calculated as LVESP/(LVESV – $V_{0}$) = LVESP/LVESV, $V_{0}$ is the left ventricular volume when the end-systolic pressure is 0. Ea was calculated as LVESP/SV and the VVI was calculated as Ea/Ees [24].

Transthoracic acquisitions of three consecutive long-axis images (apical four-chamber, three-chamber, and two-chamber) were performed during image capture, with careful attention paid to the absence of arrhythmia during image capture. Quantitative analysis software was used for off-line analysis. Specifically, the endocardial border of the apical four-chamber, two-chamber, and three-chamber views was manually traced and adjusted at end-systole. Longitudinal strain curves were automatically processed and the LVGLS value from all three views was calculated. Negative strain values indicate left ventricular myofiber shortening. To ensure the accuracy and reliability of the measurements, LVGLS was measured independently by two experienced doctors (WL with 10 years of experience, and XL with 12 years of experience) in a blinded fashion and without knowledge of the subjects’ information.

### 2.3 AVI

**Fig. 1.**
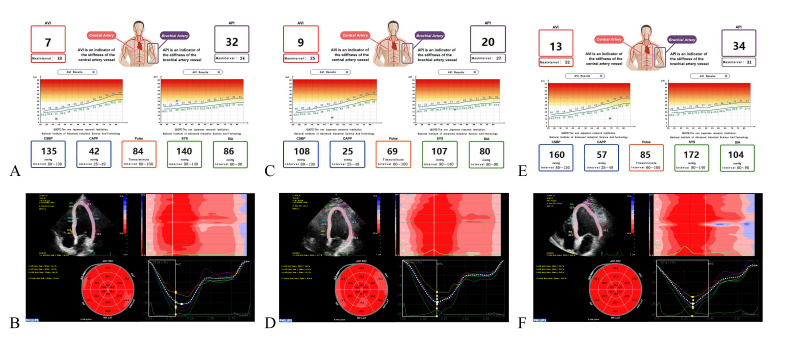
**Analysis of AVI and LVGLS**. (A,B) Male, 39 years, AVI = 7, LVGLS = –21.5%, AVI/LVGLS = –32.56. (C,D) Female, 48 years, AVI = 9, LVGLS = –22.4%, AVI/LVGLS = –40.18. (E,F) Female, 65 years, AVI = 13, LVGLS = –24.9%, AVI/LVGLS = –34–52.21. AVI, arterial velocity pulse index; LVGLS, left ventricular global longitudinal strain; API, arterial pressure volume index; CSBP, central systolic blood pressure; CAPP, central artery pulse pressure; SYS, systolic; DIA, diastolic.

The AVI was obtained using PASESA AVE-2000Pro (Shisei Datum, Tokyo, Japan). Participants were seated and allowed to rest for 5 minutes before placing their left upper arm on the cuff. Their name, age, gender, height, and weight information were inputted. The instrument automatically obtained the AVI after pressing the “measure” button. Each subject’s measurements were repeated at 5-minutes intervals, and the average of three measurements was calculated (Fig. 1).

The proposed VVI index was calculated as the ratio of the arterial stiffness measured by AVI, and the myocardial performance estimated with LVGLS (AVI/LVGLS ratio). This was compared with the widely used VVI calculated by echocardiography as described above.

### 2.4 Repeatability

Twenty subjects were randomly selected and AVI and LVGLS were measured by two physicians for inter-group repeatability. One week later, AVI and LVGLS measurements were repeated by one of the physicians on the same 20 subjects to assess intra-group repeatability.

### 2.5 Statistical Analysis

SPSS 23.0 (IBM, Armonk, NY, USA) statistical software was used for statistical analyses. Normality and homogeneity of variance were tested for quantitative data, expressed as the mean and standard deviation. Comparison of the means between two groups was performed using *t*-test, while comparison of the means among three groups was performed using one-way analysis of variance. Pairwise comparison of means between groups was conducted using LSD-*q* test. Qualitative data were represented as examples, and the chi-square test was used for comparison. Pearson correlation analysis was used for univariate correlation analysis, and multiple stepwise linear regression analysis was used for multivariate correlation analysis. Bland-Altman and linear regression analysis were used to assess the repeatability of AVI and LVGLS measurements. The diagnostic efficacy of AVI and Ea, LVGLS and Ees, AVI/LVGLS and VVI for differentiating changes in cardiovascular function in subjects from different age groups was evaluated by ROC curve. A two-sided *p*-value of <0.05 was considered statistically significant in all analyses.

## 3. Results

### 3.1 Comparison of Novel Index of Ventricular-Vascular Coupling between Subjects of Different Ages

A total of 239 subjects were included in this study, consisting of 109 males (45.6%) and 130 females (54.4%) with a mean age of 46.16 ± 13.51 years.

The general factors, cardiac structure and functional indicators of subjects from the different age groups are summarized in Table 1. The old group had 52 subjects, of which 48 (96.2%) were aged 60–69 years and 4 (3.8%) were aged 70–80 years. Compared to the young group, the middle-age and old groups had significantly higher Ea values, indicating increased arterial stiffness. No significant differences in Ees and VVI were observed between the different age groups. AVI increased progressively with age and the highest value was observed in the elderly group, suggesting that arterial stiffness was greatest in this group. LVGLS was lower in both the middle-age and old groups compared with the young group, indicating impaired myocardial function. The AVI/LVGLS ratio was smallest in the young group, followed by the middle-aged and then the old group. The difference in AVI/LVGLS ratio between the three groups was statistically significant, suggesting it can identify and reflect the ventricular-vascular coupling relationship in subjects with different ages (Table 1).

**Table 1.** **Comparison of general factors, cardiac structural function, and cardiovascular coupling indices between different age groups**.

Items		Young group (n = 112)	Middle-age group (n = 75)	Old group (n = 52)	*F/$\chi^{2}$* value	*p-*value
Female (n, %)		56 (50%)	46 (61%)	28 (54%)	2.155	0.340
Height (cm)		167.15 ± 8.51	161.83 ± ${\mathrm{7.87}}^{*}$	163.60 ± ${\mathrm{7.05}}^{*}$	10.514	<0.001
Weight (kg)		64.60 ± 12.94	62.08 ± 10.53	60.81 ± 9.01	2.432	0.092
BMI (${\mathrm{kg/m}}^{2}$)		22.99 ± 3.52	23.61 ± 2.95	22.66 ± 2.53	1.544	0.216
BSA ($m^{2}$)		1.69 ± 0.20	1.63 ± ${\mathrm{0.17}}^{*}$	1.62 ± ${\mathrm{0.15}}^{*}$	3.937	0.022
Heart rate (beats/min)		75.07 ± 12.28	69.60 ± ${\mathrm{12.53}}^{*}$	70.59 ± ${\mathrm{10.07}}^{*}$	4.909	0.008
SBP (mmHg)		130.03 ± 21.10	132.45 ± 18.61	133.73 ± 18.73	0.712	0.492
DBP (mmHg)		85.42 ± 13.34	86.83 ± 13.64	79.94 ± 9.78^*#^	4.839	0.009
MAP (mmHg)		100.29 ± 14.64	102.04 ± 14.04	97.87 ± 11.45	1.396	0.250
Echocardiography						
	LVEDD (mm)	45.44 ± 3.51	45.60 ± 3.77	45.17 ± 3.85	0.218	0.804
	LVESD (mm)	28.40 ± 2.78	28.34 ± 3.26	28.42 ± 3.07	0.013	0.987
	LVEDV (mL)	75.98 ± 18.93	69.76 ± ${\mathrm{23.93}}^{*}$	66.33 ± ${\mathrm{13.61}}^{*}$	5.349	0.006
	LVESV (mL)	28.31 ± 9.27	26.83 ± 12.25	26.48 ± 7.78	0.607	0.546
	SV (mL)	47.67 ± 12.82	42.93 ± ${\mathrm{13.97}}^{*}$	39.86 ± ${\mathrm{8.81}}^{*}$	7.911	0.001
	LVM (g)	134.46 ± 31.23	135.82 ± 26.85	136.66 ± 28.29	0.113	0.893
	E/A ratio	1.58 ± 0.54	1.12 ± ${\mathrm{0.36}}^{*}$	0.96 ± ${\mathrm{0.36}}^{*}$	41.953	<0.001
	TDI *$e^{\prime }$* (cm/s)	15.26 ± 2.61	11.66 ± ${\mathrm{3.42}}^{*}$	10.00 ± 2.13^*#^	94.573	<0.001
	LVEF (%)	0.63 ± 0.07	0.62 ± 0.07	0.60 ± 0.07	1.564	0.212
Ventricular-vascular coupling						
	Ea (mmHg/mL)	2.64 ± 0.81	3.05 ± ${\mathrm{1.09}}^{*}$	3.13 ± ${\mathrm{0.75}}^{*}$	6.756	0.002
	Ees (mmHg/mL)	4.54 ± 1.55	5.37 ± ${\mathrm{2.61}}^{*}$	4.99 ± 1.85	2.900	0.060
	VVI	0.62 ± 0.19	0.63 ± 0.22	0.68 ± 0.21	1.348	0.262
	LVGLS (%)	–22.79 ± 4.07	–21.45 ± ${\mathrm{3.70}}^{*}$	–20.57 ± ${\mathrm{3.43}}^{*}$	5.189	0.006
	AVI	10.11 ± 3.24	13.69 ± ${\mathrm{5.26}}^{*}$	15.98 ± 5.01^*#^	37.098	<0.001
	AVI/LVGLS ratio	–46.76 ± 18.94	–65.84 ± ${\mathrm{32.37}}^{*}$	–80.19 ± 28.22^*#^	27.851	<0.001

Note: Young group subjects were aged 18 to 44 years; Middle-age group subjects were aged 45 to 59 years; Old group subjects were aged 60 to 80 years. Echocardiography, the index of left ventricular structure and function were obtained by Echocardiography; Ventricular-vascular coupling, the traditional and novel index system of ventricular-vascular coupling; BMI, body mass index; BSA, body surface area; SBP, systolic blood pressure; DBP, diastolic blood pressure; MAP, mean arterial pressure; LVEDD, left ventricle end-diastolic dimension; LVESD, left ventricle end-systolic dimension; LVEDV, left ventricle end-diastolic volume; LVESV, left ventricle end-systolic volume; SV, stroke volume; LVM, left ventricular mass; E/A, E and A mitral inflow waves by Doppler; TDI, Tissue Doppler Imaging; *$e^{\prime }$*, early diastolic velocity of the mitral annulus by TDI; LVEF, left ventricular ejection fraction; Ea, effective arterial elastance; Ees, left ventricular end-systolic elastance; VVI, ventricular-vascular coupling index; AVI, arterial velocity pulse index; LVGLS, left ventricular global longitudinal strain. Compared with the Young group, ^*^p < 0.05; Compared with the Middle-age group, ^#^*p* < 0.05.

### 3.2 Comparison of the Novel Index for Ventricular-Vascular Coupling between Genders

We next investigated possible gender differences in AVI/LVGLS. Males showed significantly greater values for the general factors (height, weight, BMI, SBP, DBP, MAP) and left ventricular structural indices (LVEDD, LVESD, LVEDV, LVESV, SV and LVM) compared to the female subjects (Table 2). Both Ea and Ees were greater in female subjects than in male subjects. No significant gender difference was observed for VVI. Females showed higher LVGLS than males, suggesting their myocardial function was stronger than in men. No significant differences were observed between males and females for AVI and AVI/LVGLS (Table 2).

**Table 2.** **Comparison of general factors, cardiac structural function, and cardiovascular coupling indices between males and females**.

Items		Females (n = 131)	Males (n = 108)	*t* value	*p-*value
Age (years)		46.56 ± 13.52	45.40 ± 13.79	0.658	0.511
Height (cm)		159.60 ± 5.94	170.83 ± 6.42	–14.031	<0.001
Weight (kg)		56.79 ± 8.22	70.33 ± 10.58	–10.863	<0.001
BMI (${\mathrm{kg/m}}^{2}$)		22.31 ± 2.87	24.07 ± 3.23	19.817	<0.001
BSA ($m^{2}$)		1.55 ± 0.13	1.79 ± 0.16	–12.889	<0.001
Heart rate (beats/min)		72.27 ± 11.56	72.88 ± 12.92	–0.378	0.706
SBP (mmHg)		129.04 ± 19.18	134.49 ± 20.23	–2.126	0.035
DBP (mmHg)		81.71 ± 12.19	88.09 ± 13.01	–3.895	<0.001
MAP (mmHg)		97.48 ± 13.11	103.56 ± 14.01	–3.444	0.001
Echocardiography					
	LVEDD (mm)	43.96 ± 3.18	47.20 ± 3.40	–7.610	<0.001
	LVESD (mm)	26.97 ± 2.18	30.09 ± 2.95	–9.117	<0.001
	LVEDV (mL)	65.47 ± 17.49	78.43 ± 20.97	–4.667	<0.001
	LVESV (mL)	24.43 ± 9.01	30.68 ± 10.36	–4.475	<0.001
	SV (mL)	41.05 ± 11.31	47.75 ± 13.57	–3.683	<0.001
	LVM (g)	122.48 ± 22.85	150.77 ± 28.52	–8.336	<0.001
	E/A ratio	1.33 ± 0.59	1.26 ± 0.45	1.063	0.289
	TDI *$e^{\prime }$* (cm/s)	13.12 ± 3.79	12.97 ± 3.44	0.314	0.753
	LVEF (%)	0.63 ± 0.07	0.61 ± 0.07	1.718	0.087
Ventricular-vascular coupling					
	Ea (mmHg/mL)	3.05 ± 0.94	2.73 ± 0.90	2.329	0.021
	Ees (mmHg/mL)	5.42 ± 2.24	4.40 ± 1.72	3.519	0.001
	VVI	0.61 ± 0.21	0.66 ± 0.20	–1.634	0.104
	LVGLS (%)	–22.58 ± 3.95	–20.89 ± 3.61	–3.060	0.003
	AVI	12.35 ± 5.05	12.73 ± 4.90	–0.589	0.556
	AVI/LVGLS ratio	–57.49 ± 26.63	–65.33 ± 32.22	1.825	0.070

Note: Echocardiography, the index of left ventricular structure and function were obtained by Echocardiography; Ventricular-vascular coupling, the traditional and novel index system of ventricular-vascular coupling; BMI, body mass index; BSA, body surface area; SBP, systolic blood pressure; DBP, diastolic blood pressure; MAP, mean arterial pressure; LVEDD, left ventricle end-diastolic dimension; LVESD, left ventricle end-systolic dimension; LVEDV, left ventricle end-diastolic volume; LVESV, left ventricle end-systolic volume; SV, stroke volume; LVM, left ventricular mass; E/A, E and A mitral inflow waves by Doppler; TDI, Tissue Doppler Imaging; *$e^{\prime }$*, early diastolic velocity of the mitral annulus by TDI; LVEF, left ventricular ejection fraction; Ea, effective arterial elastance; Ees, left ventricular end-systolic elastance; VVI, ventricular-vascular coupling index; AVI, arterial velocity pulse index; LVGLS, left ventricular global longitudinal strain.

### 3.3 Comparison of Novel Index of Ventricular-Vascular Coupling between Subjects with Different BMI

To investigate the impact of overweight/obesity on ventricular-vascular coupling, we divided subjects into two groups according to a BMI threshold of 24, namely an overweight/obese group (BMI ≥24) and a control group (BMI <24). The overweight/obese group had significantly higher values for general factors (height, weight, BSA, DBP and MAP) and left ventricular structural indexes (LVEDD, LVESD, LVED, LVESV, SV and LVM) compared to the control group (Table 3). However, no significant differences were observed between the two BMI groups for traditional VVIs (Ea, Ees and VVI) and for novel VVIs (AVI, LVGLS and AVI/LVGLS) (Table 3).

**Table 3.** **Comparison of general factors, cardiac structural function and cardiovascular coupling indices between subjects with different BMI**.

Items		Control group	Overweight/Obese group	*t *value	*p*-value
		(n = 157)	(n = 82)		
Age (years)		46.13 ± 14.06	45.85 ± 12.83	0.151	0.880
Height (cm)		163.80 ± 8.06	166.34 ± 8.60	–2.259	0.025
Weight (kg)		57.24 ± 7.68	73.77 ± 9.76	–13.328	<0.001
BSA ($m^{2}$)		1.58 ± 0.14	1.81 ± 0.17	–11.018	<0.001
Heart rate (beats/min)		73.38 ± 12.04	70.99 ± 12.32	1.375	0.170
SBP (mmHg)		129.81 ± 18.26	134.83 ± 22.25	–1.860	0.064
DBP (mmHg)		83.12 ± 12.59	87.49 ± 13.20	–2.493	0.013
MAP (mmHg)		98.68 ± 12.98	103.27 ± 14.97	–2.447	0.015
Echocardiography					
	LVEDD (mm)	44.52 ± 3.51	47.16 ± 3.29	–5.640	<0.001
	LVESD (mm)	27.68 ± 2.84	29.72 ± 2.81	–5.296	<0.001
	LVEDV (mL)	68.71 ± 17.76	76.76 ± 23.42	–2.662	0.008
	LVESV (mL)	26.09 ± 19.51	29.68 ± 110.91	–2.356	0.019
	SV (mL)	42.62 ± 11.35	47.08 ± 14.88	–2.131	0.035
	LVM (g)	127.01 ± 25.37	151.07 ± 29.54	–6.574	<0.001
	E/A ratio	1.32 ± 0.57	1.26 ± 0.44	0.785	0.433
	TDI *$e^{\prime }$* (cm/s)	13.24 ± 3.83	12.70 ± 3.19	1.142	0.255
	LVEF (%)	0.62 ± 0.07	0.61 ± 0.07	0.734	0.464
Ventricular-vascular coupling					
	Ea (mmHg/mL)	2.94 ± 0.88	2.83 ± 1.02	0.727	0.468
	Ees (mmHg/mL)	5.12 ± 2.15	4.60 ± 1.88	1.668	0.097
	VVI	0.63 ± 0.21	0.65 ± 0.18	–0.747	0.456
	LVGLS (%)	–22.13 ± 3.80	–21.16 ± 3.99	–1.651	0.100
	AVI	12.12 ± 4.95	13.30 ± 4.97	–1.731	0.085
	AVI/LVGLS ratio	–58.60 ± 27.65	–66.12 ± 32.60	1.660	0.099

Note: Control group were subjects with BMI <24; overweight/obese group were subjects with BMI ≥24. Echocardiography, the index of left ventricular structure and function were obtained by Echocardiography; Ventricular-vascular coupling, the traditional and novel index system of ventricular-vascular coupling; BSA, body surface area; SBP, systolic blood pressure; DBP, diastolic blood pressure; MAP, mean arterial pressure; LVEDD, left ventricle end-diastolic dimension; LVESD, left ventricle end-systolic dimension; LVEDV, left ventricle end-diastolic volume; LVESV, left ventricle end-systolic volume; SV, stroke volume; LVM, left ventricular mass; E/A, E and A mitral inflow waves by Doppler; TDI, Tissue Doppler Imaging; *$e^{\prime }$*, early diastolic velocity of the mitral annulus by TDI; LVEF, left ventricular ejection fraction; Ea, effective arterial elastance; Ees, left ventricular end-systolic elastance; VVI, ventricular-vascular coupling index; AVI, arterial velocity pulse index; LVGLS, left ventricular global longitudinal strain; BMI, body mass index.

### 3.4 Factors Affecting the AVI/LVGLS

**Fig. 2.**
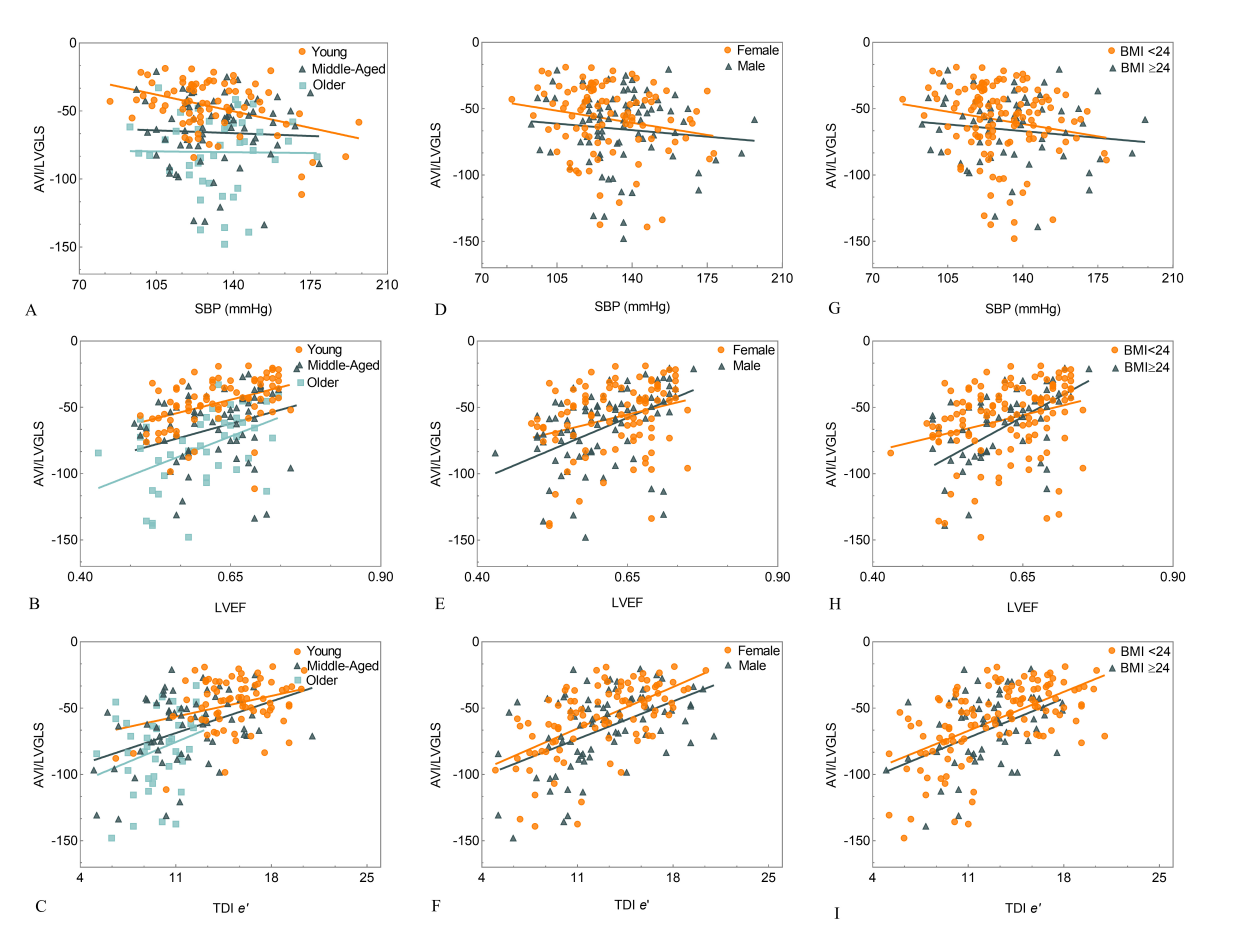
**Correlation between the AVI/LVGLS ratio and SBP, LVEF, TDI *$e^{\prime }$* in different age, gender and BMI subgroups**. AVI/LVGLS was negatively correlated with SBP (A), and positively correlated with LVEF (B) and TDI *$e^{\prime }$* (C) in different age groups. AVI/LVGLS did not correlate with SBP (D), but was positively correlated with LVEF (E) and TDI *$e^{\prime }$* (F) in different gender groups. AVI/LVGLS was positively correlated with SBP (G), LVEF (H) and TDI *$e^{\prime }$* (I) in different BMI groups. AVI, arterial velocity pulse index; LVGLS, left ventricular global longitudinal strain; SBP, systolic blood pressure; LVEF, left ventricular ejection fraction; TDI, Tissue Doppler Imaging; *$e^{\prime }$*, early diastolic velocity of the mitral annulus by TDI; BMI, body mass index.

As shown in Fig. 2A, a significant inverse relationship was observed between AVI/LVGLS and SBP in young subjects (r = –0.399, *p* < 0.05), but not in middle-age and old subjects (r = –0.034 and –0.011, respectively; both *p* > 0.05). Furthermore, AVI/LVGLS was positively correlated with LVEF in all three age groups (r = 0.428, 0.266 and 0.455, respectively; all *p* < 0.05). A positive association was also observed between AVI/LVGLS and TDI *$e^{\prime }$* in the young and the middle-age groups (r = 0.308 and 0.331, respectively; *p* < 0.05), but not in the old group (r = 0.297; *p* > 0.05) (Fig. 2A–C).

No significant correlation was found between AVI/LVGLS and SBP in either males or females (r = –0.086 and –0.193, respectively;* p >* 0.05). However, AVI/LVGLS showed a positive correlation with LVEF in both males and females (r = 0.424 and 0.286, respectively;* p <* 0.05), and also with TDI *$e^{\prime }$* (r = 0.437 and 0.596, respectively; *p* < 0.05) (Fig. 2D–F).

AVI/LVGLS was not significantly correlated with SBP in either the control or overweight/obese groups (r = –0.171 and –0.103, respectively; *p* > 0.05), but was positively correlated with LVEF (r = 0.290 and 0.503, respectively; *p<*0.05) and with TDI *$e^{\prime }$* (r = 0.579 and 0.363, respectively; *p* < 0.05) in both BMI groups (Fig. 2G–I).

In all subjects, AVI/LVGLS was negatively correlated with age, SBP, LVEDV, LVESV, Ea and VVI, (r = –0.454, –0.153, –0.149, –0.323, –0.132 and –0.371, respectively; all *p* < 0.05), and positively correlated with LVEF, E/A, TDI *$e^{\prime }$* and Ees (r = 0.367, 0.361, 0.504 and 0.146, respectively; *p* < 0.05). Age, LVEDV, LVESV and TDI *$e^{\prime }$* were identified as factors that influence AVI/LVGLS (β = –0.482, –1.492, 2.953 and –8.365, respectively; *p* < 0.05) (Table 4).

**Table 4.** **Univariable and multivariable predictors of AVI/LVGLS**.

Variables	Univariable		Multivariable			
	*r*	*p*-value	Regression coefficient (β)	Standardized coefficients ($\beta^{\prime }$)	95% Confidential interval	*p*-value
Age	–0.454	<0.001	–0.482	–0.215	(–0.817–0.147)	0.005
BMI	–0.120	0.102	–0.035	-	-	0.537
Heart rate	0.007	0.921	–0.036	-	-	0.539
SBP	–0.153	0.037	–0.016	-	-	0.795
MAP	–0.062	0.403	–0.003	-	-	0.963
LVEF	0.367	<0.001	–0.068	-	-	0.491
E/A	0.361	<0.001	0.010	-	-	0.888
LVEDV	–0.149	0.042	–0.105	-	-	0.512
LVESV	–0.323	<0.001	–1.492	–0.509	(–1.857 – –1.127)	<0.001
TDI *$e^{\prime }$*	0.504	<0.001	2.953	0.350	(1.709–4.198)	<0.001
Ea	–0.132	0.072	–8.365	–0.261	(–12.466 – –4.265)	<0.001
Ees	0.146	0.046	–0.088	-	-	0.405
VVI	–0.371	<0.001	0.097	-	-	0.338

Note: BMI, body mass index; SBP, systolic blood pressure; MAP, mean arterial pressure; LVEF, left ventricular ejection fraction; E/A, E and A mitral inflow waves by Doppler; LVEDV, left ventricle end-diastolic volume; LVESV, left ventricle end-systolic volume; TDI, Tissue Doppler Imaging; *$e^{\prime }$*, early diastolic velocity of the mitral annulus by TDI; Ea, effective arterial elastance; Ees, left ventricular end-systolic elastance; VVI, ventricular-vascular coupling index; AVI, arterial velocity pulse index; LVGLS, left ventricular global longitudinal strain.

### 3.5 Diagnostic Efficacy of Novel Indicators of Ventricular-Vascular Coupling

**Fig. 3.**
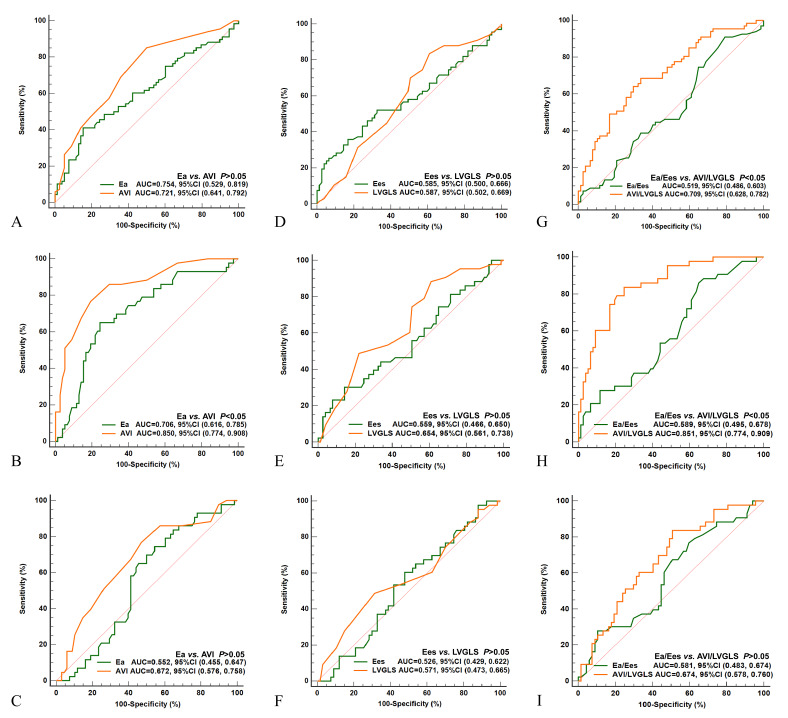
**ROC curves for the parameters of arterial and ventricular coupling**. ROC curves for Ea and AVI in young and middle-age groups (A), young and old groups (B), and middle-age and old groups (C). ROC curves for Ees and LVGLS in young and middle-age groups (D), young and old groups (E), and middle-age and old groups (F). ROC curves for VVI and AVI/LVGLS in young and middle-age groups (G), young and old groups (H), and middle-age and old groups (I). Ea, effective arterial elastance; Ees, left ventricular end-systolic elastance; AVI, arterial velocity pulse index; LVGLS, left ventricular global longitudinal strain; VVI, ventricular-vascular coupling index; ROC, receiver operating characteristic curve; AUC, area under curve.

AVI/LVGLS had significantly superior diagnostic performance compared to VVI in young and middle-age subjects, with areas under the ROC curve of 0.709 and 0.519, respectively (*p* < 0.05) (Fig. 3G). However, no significant difference in diagnostic performance was found between AVI and Ea in young and middle-age subjects (0.721 and 0.754, respectively; *p* > 0.05; Fig. 3A), nor between LVGLS and Ees (0.587 and 0.585, respectively; *p* > 0.05; Fig. 3D).

In the young and elderly subjects, the diagnostic efficacy of AVI/LVGLS was higher than that of VVI (Fig. 3H), while that of AVI was higher than Ea (Fig. 3B). There was no significant difference in diagnostic efficacy between LVGLS and Ees (Fig. 3E).

In middle-age and old subjects, no significant differences in diagnostic efficacy were found between AVI/LVGLS and VVI, AVI and Ea, and LVGLS and Ees (all *p* > 0.05) (Fig. 3C,F,I, respectively).

### 3.6 Repeatability

**Fig. 4.**
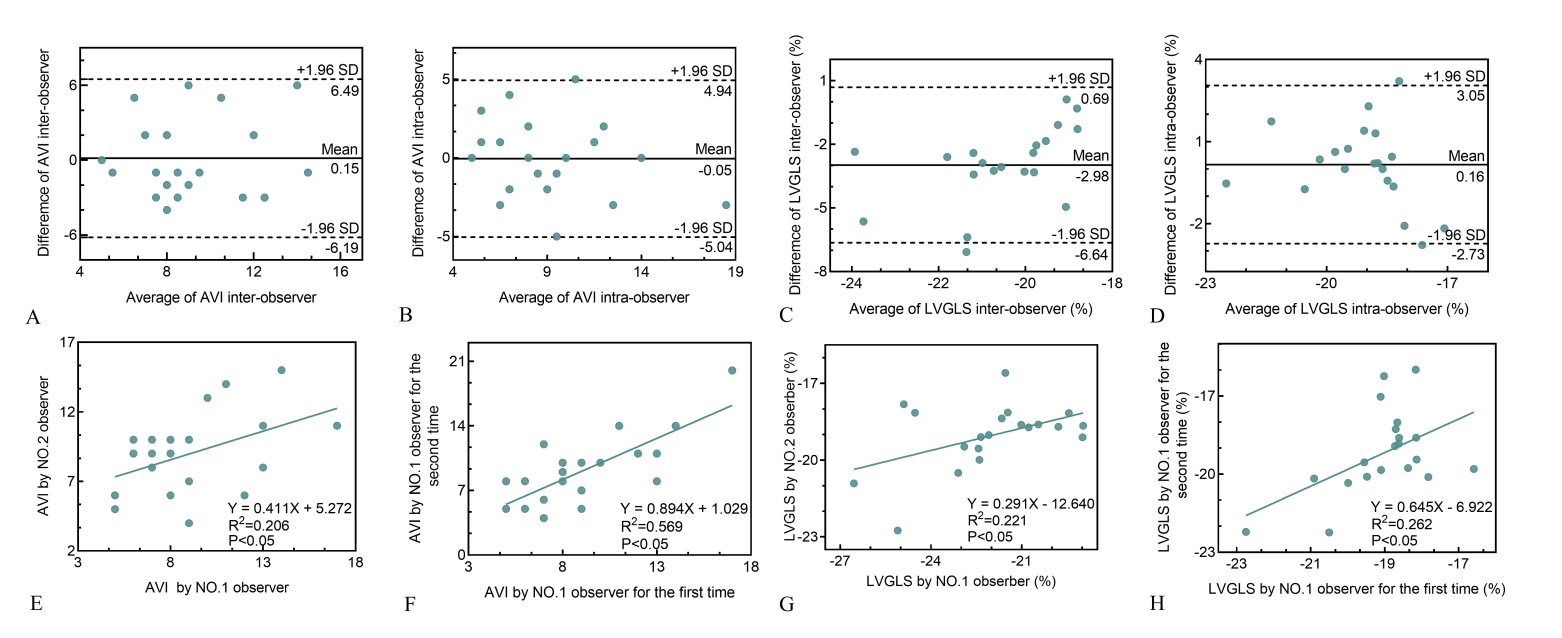
**Repeatability test of AVI and LVGLS by Bland-Altman plot and linear correlation analysis**. Bland-Altman analysis (A,B) showed a consistent trend and linear regression analysis (E,F) showed good agreement for AVI inter-observer and intra-observer respectively. Bland-Altman analysis (C,D) showed a consistent trend and linear regression analysis (G,H) showed good agreement for LVGLS inter-observer and intra-observer respectively. AVI, arterial velocity pulse index; LVGLS, left ventricular global longitudinal strain.

The repeatability of AVI was evaluated by inter- and intra-group comparisons. These revealed a high consistency in the measurements [between: $R^{2}$ = 0.206, *p* < 0.05, mean difference (0.15 ± 3.06); within groups: $R^{2}$= 0.569, *p* < 0.05, mean difference (–0.05 ± 2.55)]. The Bland-Altman plots indicated the 95% limits of agreement were –6.19–6.49 for inter-observer, and –5.04–4.94 for intra-observer comparisons (Fig. 4).

Similarly, the repeatability of LVGLS was assessed by between- and within-group comparisons. These revealed a high consistency in the measurements [between: $R^{2}$ = 0.221, *p* < 0.05, mean difference (–2.98 ± 1.87) %; within groups: $R^{2}$ = 0.262, *p* < 0.05, mean difference (0.16 ± 1.47) %]. Bland-Altman analysis further confirmed the high consistency of LVGLS repeat measurements (Fig. 4).

## 4. Discussion

### 4.1 Summary

The main findings of this study were: (1) AVI, LVGLS, and AVI/LVGLS showed age-related changes, with AVI and AVI/LVGLS increasing with age and LVGLS decreasing. Our study suggests that arterial stiffness increases with age, while left ventricular systolic function decreases and the ratio of the two increases, thereby reflecting age-related changes in the cardiovascular system. AVI/LVGLS was found to increase with age and correlated inversely and independently with age. LVESV and Ea were positively correlated with TDI *$e^{\prime }$*, which is an indicator of left ventricular diastolic function. (2) Males had lower Ea, Ees and LVGLS than females, while AVI and AVI/LVGLS did not differ between the genders. (3) AVI, LVGLS and AVI/LVGLS are novel VVI systems that are more effective than traditional indexes at detecting differences in cardiovascular function in different age groups.

### 4.2 AVI and Ea

AVI provides a more direct assessment than Ea of the total elastance of the arterial tree and the resistance of peripheral arteries, both of which are closely related to cardiac function. The present study revealed that the novel arterial stiffness index AVI was more sensitive than Ea in identifying the degree of arterial aging in subjects with different ages. Furthermore, it was more stable in its assessment of differences in arterial elastance between genders. Arterial stiffness increases with age [9] and is also promoted by various factors associated with aging, such as increased arterial wall stress, oxidative stress, and inflammatory stimuli. These factors decrease the elastic fibers in arteries that maintain vascular compliance, whereas the collagen fibers increase. This leads to changes in the structure and function of the arterial wall, resulting in increased vascular stiffness and decreased compliance [25, 26]. AVI, a novel index of arterial stiffness, was found in this study not only to reflect the stiffness of the central artery, but also to correlate significantly with total peripheral vascular impedance and left ventricular contractility [15]. Previous studies have shown that AVI is a sensitive predictor of the risk of subclinical coronary atherosclerosis without significant obstruction in the lumen [22]. Furthermore, AVI is independently associated with the concentration of plasma atrial natriuretic peptide and with a history of congestive heart failure [27], suggesting that enhanced AVI may reflect an increase in cardiac workload. The present study also demonstrated that Ea could differentiate vascular elastance between young and middle-age individuals, but not between middle-age and old individuals. This result suggests that Ea has limitations for evaluating the elastance of arteries in middle-age and elderly people. Ea reflects the elastance of the arterial tree and the resistance of peripheral vessels, as well as reflecting the overall afterload of the heart [4]. It is determined by the formula Ea = LVESP/SV, which reflects only the reserve function of the heart SV [24]. Ea may have reduced diagnostic efficacy in middle-age and elderly individuals due to a decrease in cardiac stroke reserve [9]. Additionally, the inadequate assessment of systemic common artery elastance by Ea limits its clinical application [24].

### 4.3 LVGLS and Ees

LVGLS has greater sensitivity than Ees for the evaluation of impaired cardiac systolic function [5]. Of note, LVGLS has the ability to differentiate cardiac function across different age groups, as well as between genders. Various studies have confirmed that LVGLS enables earlier detection of impaired systolic function and can thus function as an early warning for cardiovascular and cerebrovascular events [28, 29]. LVGLS, as a contrasting predictor of cardiovascular events, has been incorporated into guidelines for the early assessment of cardiac ventricular disorders [30]. Reduced LVGLS serves as a partial manifestation of impaired systolic function in patients with HFpEF [31]. Moreover, a follow-up study of 4172 patients with acute heart failure revealed that LVGLS alone was an independent predictor of all-cause mortality at 5 years, regardless of whether or not LVEF was normal [32]. Myocardial compliance decreases as cardiomyocytes undergo apoptosis or necrosis with aging and as the extracellular matrix undergoes fibrotic remodeling, thus leading to functional changes [33]. The present study found that Ees was higher in middle-age and old subjects compared to young subjects. Ees reflects ventricular contractility and systolic stiffness, and is an essential parameter for assessing cardiac systolic function and hemodynamic status. However, Ees does not take into account myocardial geometry and tissue structure (such as cardiomyocytes and elastic fibers), both of which have a significant impact on ventricular systolic and diastolic function [6]. The present study found that ventricular end-diastolic volume decreased with increasing age, while the ejection fraction remained unchanged. This finding explains why Ees behaves differently across different age groups and in obese individuals.

### 4.4 AVI/LVGLS and VVI 

An increased AVI is indicative of increased arterial stiffness, while decreased absolute LVGLS values suggest impaired ventricular myocardial systolic function and are also associated with diastolic dysfunction [31]. The present study examined the interaction between AVI and LVGLS, which represents ventricular-vascular interactions. Possible reciprocal mechanisms include increased arterial stiffness, decreased peripheral vascular compliance, increased ventricular afterload, decreased coronary perfusion and myocardial oxygen delivery. These result in cardiomyocyte hypertrophy, fibroblast growth and interstitial fibrosis, leading to ventricular hypertrophy, and systolic and diastolic dysfunction [7]. Based on this, the AVI/LVGLS ratio is closely associated with left ventricular diastolic function indicators such as E/A and TDI *$e^{\prime }$*. We found no significant differences in VVI between different age groups, whereas AVI/LVGLS gradually increased with age. Higher AVI was associated with higher vascular stiffness in the elderly, which may be accompanied by subclinical left ventricular dysfunction. This leads to lower LVGLS and subsequently to an increased AVI/LVGLS ratio. In addition, we compared the diagnostic performance of AVI/LVGLS and traditional VVI in different age groups. AVI/LVGLS was found to have high area under curve (AUC), sensitivity and specificity, and a superior diagnostic performance to that of VVI. Therefore, AVI/LVGLS is a valuable tool for early detection of subclinical cardiac-vascular coupling mismatch.

It is worth noting that the AVI/LVGLS ratio, a novel index system for ventricular-vascular coupling, is consistent with previous VVI obtained through echocardiography. Moreover, in healthy populations it does not differ significantly according to gender or BMI. There are several potential explanations for this observation. Firstly, overweight/obese individuals and those with metabolic diseases may have impaired subclinical myocardial contractility which can affect LVGLS [30]. Studies have shown that bariatric surgery can improve LVGLS in severely obese individuals (BMI ≥35), and that subclinical myocardial dysfunction is associated with visceral adiposity and inflammatory markers rather than with BMI or fat mass [34, 35]. Secondly, gender differences may play a role, as LVGLS is typically higher in females than males. This is due to more deformation of the smaller LVM in females, resulting in increased intraventricular blood flow efficiency and decreased energy expenditure [36, 37]. In contrast, males may compensate for relatively low blood flow efficiency by increasing kinetic energy. Thus, the interaction between arteries and ventricles as reflected by the AVI/LVGLS ratio appears to conform to physiological characteristics and is not solely determined by numerical values.

## 5. Limitations

There are several limitations to this study. First, this was an exploratory study aimed at establishing the basic clinical value of the cardiac-vascular coupling index system. It was therefore a preliminary study conducted on the normal population, and its application in various diseases has yet to be investigated. We plan to conduct further research on the characteristics of these indexes in patient cohorts with common diseases such as diabetes and hypertension. Second, the VVI measurement in this study was obtained by echocardiography rather than by using the gold standard invasive pressure-volume curve loop. Our novel index system for ventricular-vascular coupling is non-invasive, simple, and highly applicable for extensive clinical work and large-scale community screening. Third, our novel index system was not compared with previous PWV/LVGLS, which we plan to investigate in future studies.

## 6. Conclusions

The novel cardiac-vascular coupling index system described in this study is more effective at identifying age-related changes in cardiovascular disease compared to the traditional system. Moreover, it is not affected by gender or overweight/obesity. Among healthy individuals, the AVI/LVGLS index is a more sensitive and reliable diagnostic tool than VVI for assessing cardiac-vascular couplings at different ages, and is independently associated with left ventricular diastolic dysfunction. The use of AVI, LVGLS and AVI/LVGLS indexes may provide a straightforward and reproducible way to evaluate cardiovascular function status. Moreover, these indexes could offer new perspectives for investigating cardiovascular reserve function in patients with various diseases.

## Data Availability

All data generated or used during the study appear in the submitted article.
